# *Lannea coromandelica* (Houtt.) Merr.: A Comprehensive Review of Its Ethnomedicinal Uses, Phytochemistry and Pharmacological Activity

**DOI:** 10.1002/hsr2.71752

**Published:** 2026-01-18

**Authors:** Nawfal Hasan Siam, Sumaya Akter, Anika Rahman, Taniza Tasnim, Md. Mhamudul Huda Ruhed, Anika Ahmed, Naznin Rahman, Afsana Akter Mim, Tasnim Tabassum, Md. Mazharul Islam Chowdhury, Jakir Ahmed Chowdhury

**Affiliations:** ^1^ Department of Pharmacy School of Pharmacy and Public Health, Independent University, Bangladesh, (IUB) Dhaka Bangladesh; ^2^ Department of Medical Biotechnology, Faculty of Science University of Technology Sydney (UTS) Sydney New South Wales Australia; ^3^ Department of Public Health & Informatics Jahangirnagar University, Savar Dhaka Bangladesh; ^4^ Department of Pharmaceutical Sciences Appalachian College of Pharmacy Oakwood Virginia USA; ^5^ Department of Pharmaceutical Technology, Faculty of Pharmacy University of Dhaka Dhaka Bangladesh

**Keywords:** active compound, analgesic, anticancer, antidiabetic, anti‐inflammatory, antioxidant

## Abstract

**Background and Aims:**

*Lannea coromandelica* (Houtt.) Merr., a deciduous tropical tree of the Anacardiaceae family, is traditionally used in Asian ethnomedicine to treat diabetes, skin diseases, ulcers, inflammation, and microbial infections. This review aims to consolidate current knowledge on its ethnomedicinal applications, phytochemistry, pharmacology, and toxicology to assess its therapeutic potential.

**Methods:**

A systematic literature search was conducted from January to April 2025 using Web of Science, PubMed, Science Direct, and Google Scholar. Approximately 115 papers were initially screened, with 86 selected for comprehensive review. The data covered publications from 2011 to 2025, focusing on keywords related to *L. coromandelica* and associated terms.

**Results:**

Phytochemical studies revealed diverse bioactive constituents, including flavonoids (quercetin, morin), phenolic acids (gallic acid, protocatechuic acid), triterpenes (oleanolic acid, myricadiol), and sterols (β‐sitosterol). Pharmacological investigations demonstrated antidiabetic, antioxidant, anticancer, antimicrobial, anti‐inflammatory, anti‐ulcer, antihistaminic, and analgesic effects. Extracts significantly reduced hyperglycemia, improved lipid profiles, scavenged free radicals, and inhibited tumor growth in experimental models. Toxicology assessments indicated a high safety margin with no observed toxicity or mortality at doses above 3000 mg/kg in animals.

**Conclusion:**

*L. coromandelica* exhibits promising pharmacological activities and safety, highlighting its potential as a source for novel phytotherapeutics. Nonetheless, further studies on chronic toxicity, pharmacokinetics, and mechanisms of action are essential to support clinical development.

## 1 Introduction

*Lannea coromandelica* (Houtt.) Merr. is a deciduous tropical tree that is extensively found across Asia, including countries like Bangladesh, India, Sri Lanka as well as in several other tropical regions [1]. *L. coromandelica*, a member of the Anacardiaceae family, is a medicinally valuable tree widely used in indigenous traditional medicine systems [2]. It is a medium‐sized deciduous tree that can grow up to 14 m in height. It is characterized by alternately arranged pinnate leaves, typically comprising five ovate leaflets, and branches covered with distinctive star‐shaped hairs. The species is further identified by its unisexual, greenish flowers and ovoid fruits arranged in panicles, which serve as key taxonomic features [3].

Widely recognized in traditional medicine, *L. coromandelica* has been extensively employed for its therapeutic properties. The bark, leaves, and resin are traditionally valued for their astringent, antioxidant, anticancer, anti‐inflammatory, and antidiabetic activities [4]. It has long been used to manage various health conditions, including skin disorders such as eczema and psoriasis, digestive ailments, and arthritis. In folk medicine, species of the *Lannea* genus are also utilized in the treatment of elephantiasis, impotence, vaginal infections, halitosis, heart disease, dysentery, gout, and rheumatism. Traditionally, the bark and leaves are frequently used to alleviate a variety of symptoms. The juice extracted from the leaves has been consumed orally to relieve ulcers and pain [3, 5]. Despite its wide range of ethnomedicinal uses, phytochemical investigations of the Lannea genus remain limited. However, several bioactive compounds such as Glycosides, Terpenoids, Polyphenols, flavonoids, hydroquinones, and ferulic acid esters have been isolated. L. coromandelica has demonstrated numerous pharmacological activities [4], including hypotensive, antidiabetics, antibacterial, anti‐inflammatory, antimicrobial, antinociceptive, Antitubercular activity, anxiolytic, anti‐Ulcer, antithelmintic, hypolipidemic activity, wound‐healing, anticancer, Antidiarrheal Activity and *in vitro* antifilarial effects. Phytochemical analyses have revealed the presence of diverse constituents such as phenolic compounds, flavonoids, triterpenoids, tannins, and alkaloids [2]. Over the past few decades, extensive research has been devoted to exploring the ethnobotanical, pharmacological [6, 7], and phytochemical characteristics of *L. coromandelica*. Recent investigative trends underscore its significant potential as a source of novel therapeutic agents derived from its diverse phytochemical profile and bioactivities. To date, however, no comprehensive review has systematically consolidated its traditional applications, chemical constituents, and pharmacological effects. In response to this gap, the present review aims to provide a detailed synthesis of the ethnomedicinal uses, phytochemical composition, pharmacological properties, and safety aspects of *L. coromandelica*. This compilation is intended to serve as a valuable resource and reference framework to support and guide future drug discovery endeavors centered on this promising medicinal plant.

## 2 Methodology

**Figure 1:**
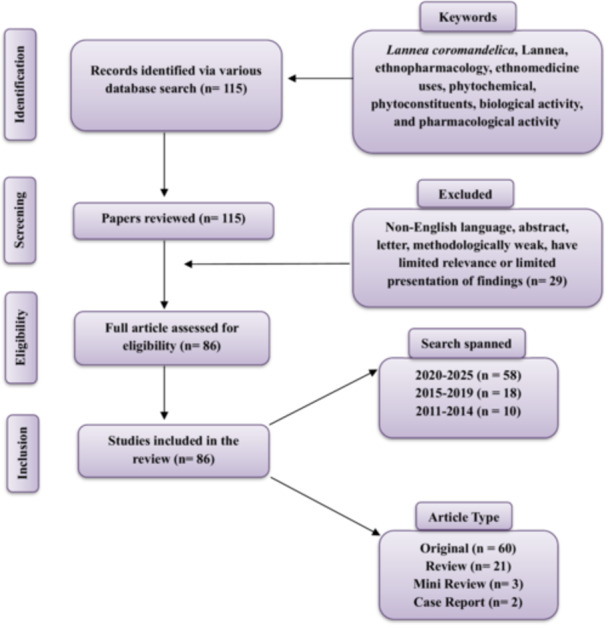
Methodological approach utilized for the literature survey conducted in this review.

Information for this review was collected from Web of Science, PubMed, ScienceDirect, and Google Scholar using specific keywords, including *Lannea coromandelica*, *Lannea*, ethnopharmacology, ethnomedicine, phytochemical, phytoconstituents, biological activity, and pharmacological activity. The search covered the period from 2011 to 2025. Data collection was conducted between January and April 2025, during which approximately 115 papers were screened to identify relevant studies. After initial screening, 86 papers were selected for detailed examination and inclusion in this review. A concise overview of the literature matrix and the selection criteria applied is presented in Figure 1.

## 3 Vernacular Name and Taxonomy

Vernacular or local names are essential for recording and interpreting biodiversity, as they capture the cultural and linguistic connections between people and the natural world (Table 1). They complement scientific names by preserving traditional ecological knowledge and cultural heritage. The local names of these species from different regions are summarized in Table 1.

**Table 1:** Vernacular names of L. coromandelica in various countries.

Country	Vernacular names	Ref
Bangladesh	Jiga, badi, kapula, bhadi, bohar, ghadi, jail, jial bhandi, jigor, jiol, jir, jival, kasmala, lohar, jika, jiga gach, jibli gach	[8, 9]
Cambodia	Pon teuk	[10]
India	Jhingan, jhingan, jhingan mohin, godda mara, udi mara, ajjashringi, uthian, mohin, moi, kondh tribe‐kanbeli, poraja, dumpidi, shimiti, annakara, gholdo.	[11, 12, 13, 14, 15, 16],
Indonesia	kayu jawa, pohon kudo,	[17, 18, 19]
Myanmar	Maing	[14]
Nepal	Thulo dabdabe, Dabdabe, Jangra	[14, 20]
Pakistan	Kembal, kalmaan	[14] [21]
Sri Lanka	Hik	[22]
Thailand	Oi Chang	[23]

**Figure 2:**
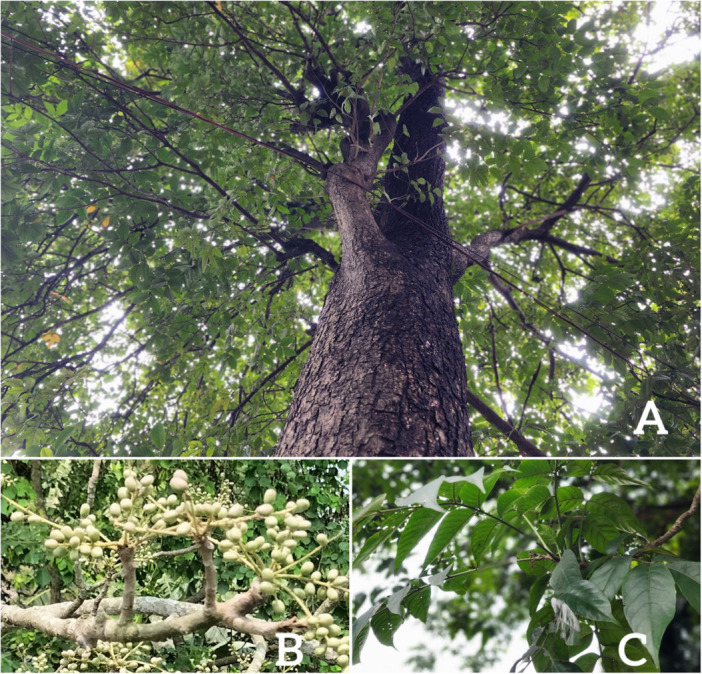
L. coromandelica. A: Full plant, B: Fruits, C Leaves.

*L. coromandelica* is a deciduous tree native to South and Southeast Asia. It is widely distributed across Bangladesh, India, China, Nepal, Sri Lanka, Pakistan, Malaysia, Myanmar, Thailand, and Vietnam, as well as in specific regions such as the Andaman Islands, Assam, Cambodia, Guangdong, Guangxi, Hainan, Laos, and Yunnan. It typically reaches a height of 5–14 m. It is distinguished by its gray‐white bark and thick branchlets, which are densely covered with rust‐colored stellate hairs. The leaves are odd‐pinnate, usually clustered at the ends of branchlets, measuring between 10 and 33 cm in length. They commonly bear 7–9 (occasionally 5–11) pairs of ovate leaflets, with 2 to 5 pairs along the rachis and cylindrical petioles that are sparsely covered with similar rust‐colored stellate hairs. The flowers are small, yellow or purplish in color, and are born in terminal, either branched or unbranched racemes. The fruit is an ovate drupe, slightly compressed, turning purplish‐red when mature. Each drupe measures approximately 6–10 mm in length and about 0.5 mm in width and is glabrous when ripe [24] [Figure 2]. The full taxonomic classification of *L. coromandelica* is outlined below [25].

Kingdom: Plantae

Phylum: Tracheophyta

Class: Magnoliopsida

Order: Sapindales

Familyn: Anacardiaceae

Genus: Lannea A. Rich.

Species: *Lannea coromandelica* (Houtt.) Merr.

## 4 Ethnomedicinal Uses

The plant under investigation has been utilized across several Asian countries for a wide array of ethnomedicinal purposes, with different parts of the plant including bark, leaves, roots, stem, sap, gum, fruit, and trunk being employed in traditional healing systems [Table 2]. In South and Southeast Asia, *L. coromandelica* is traditionally used for managing various chronic ailments. Macerated material or expressed juice treats piles and chronic dysentery [26], while bark or root preparations‐soaked overnight help regulate blood glucose in diabetes [27]. Leaf pastes are applied for urinary tract problems and diabetes management [28]. In China, both the leaves and bark are processed through decoction and maceration techniques. These preparations are traditionally used to treat physical injuries and hematochezia (blood in stool) [13]. Indonesia showcases a rich diversity in plant usage. The bark is decocted for internal diseases and low back pain [17], heated to dress open wounds, or pounded into a liquid to treat hematochezia. The outer bark is boiled to cure diarrhea, and bark powder is used to heal wounds in diabetic mice [29]. Ethanolic extracts from the stem are applied to monitor serum creatinine levels [30]. The leaves are typically boiled and consumed to manage fever [17], blood glucose levels [33], and as a cough and ulcer remedy, or appetite enhancer. Other forms of preparation include maceration [31] and aqueous extracts to increase erythrocyte concentration in ducks [32, 33, 36]. Decoctions are also used for fertility issues, diabetes, and hypertension [17]. Sap from the trunk, when pounded into a liquid, is applied to relieve sore eyes and fever. Decoctions of the trunk serve in treating hematemesis and tuberculosis.

**Table 2:** Ethnomedicinal uses of L. coromandelica in various countries.

Country	Part used	Preparation & method extraction	Ethnomedicinal uses	Ref
Bangladesh	Bark	Macerated and juice	Treatment of piles, chronic dysentery	[26]
	Barks or roots	Soaked in water overnight	Treatment of diabetes	[27]
	Leaves	Paste	Treatment of urinary problems, diabetes	[28]
China	Leaves and bark	Decoction and macerations	Treatment of injuries and hematochezia	[13]
Indonesia	Bark	Decoction	Treatment of low back pain and internal diseases	[17]
		Heating	Treatment of open wounds	
		Pounded to make liquid form	Treatment of hematochezia	
		Outer layer of bark is pounded and boiled	Treatment of diarrhea	
		Powder	Treatment of wounds	[29]
		Stem and ethanolic extract	Measurement serum creatinine levels	[30]
	Leaves	Boiled, and Consumed	Treatment of fever	[17]
		The maceration method	Reduce blood glucose levels	[31]
		Aqueous extract	Increases erythrocyte (red blood cell) concentrations in Peking ducks (Anas platyrhynchos).	[32]
		Boiled	Lower blood glucose level	[33]
		Decoction	Treatment of cough, ulcer, and appetite enhancer	[31]
		Boiled	Treatment of fertility disorders, diabetes, hypertension	[17]
	Sap from trunk	Pounded to make liquid form	Treatment of sore eyes and fever	
	Trunk	Decoction	Treatment of hematemesis, tuberculosis	
India	Leaves	Juice	Treatment of ulcers, tooth ache	[13]
			Antidote in coma caused by narcotic	[12]
		Boiled	Treatment of local swellings and body pains	
		Paste	Treatment of sprains, bruises, elephantiasis, inflammation, neuralgia	
	Stem bark	Paste	Treatment of body pains, applied on cuts and burns, antifertility drug, seminal weakness and excessive seminal emission	[12, 13]
		Decoction	Treatment of gastric trouble, menorrhagia and metrorrhagia	[12]
	Gum	Mixed with coconut oil	Topical application on infected areas to cure skin diseases	[34]
		Obtained from the tree′s bark and given as a cordial	Treatment of asthma, rheumatism and women during lactation	[35]
		Soaked in water, rubbed on stone	Treatment of pain	[13]
		Directly applied	Treatment of piles and wounds	[15]
	Fruits	Crushed	Treatment of fish poison	[13]
		Paste	Treatment of bone fractures	[12]
	Inner bark of stem	Crushed and make suspension juice	Stops bleeding and to prevent tetanus	[13]
	Bark	Crushed	Treatment of dysentery	[13]
		Paste	Treatment of stomach pain	
		Lotion	Treatment of leprous ulcers	
Nepal	Leaf	Juice	Treatment of cuts	[20]
Pakistan	Leaf, bark, wood	Decoction. Tea	Treatment of feet inflammations, sprains, body swellings, ulcers, elephantiasis and used as evms	[21]
Thailand	Bark		Wound dressing, Heartwood: Used to prepare a remedy for soothing the throat and relieving thirst	[23]

In India, the leaves are processed into juice to manage ulcers and toothaches [13] or boiled for relieving swellings and body pain. Paste from leaves is traditionally used to treat sprains, bruises, elephantiasis, inflammation, and neuralgia. Stem bark preparations, such as pastes and decoctions, are used for body pain, cuts and burns, antifertility treatments, seminal weakness, gastric disorders, and menstrual irregularities [12, 13]. Gum obtained from the bark is mixed with coconut oil for treating skin diseases [34], and also used in cordials for asthma, rheumatism, and to aid lactating women [35]. Other bark preparations include rubbing soaked bark on stone to relieve pain [13], direct application for piles and wounds [15], and paste or crushed forms to address dysentery, stomach pain, and leprous ulcers. The fruits are crushed as fish poison [13], and in paste form, applied to bone fractures [12]. The inner bark is crushed and suspended in juice to stop bleeding and prevent tetanus [13]. In Nepal, the leaf juice is applied topically to treat cuts [20]. In Pakistan, a decoction made from the leaf, bark, and wood is consumed as tea to alleviate feet inflammations, sprains, body swellings, ulcers, elephantiasis, and for use as ethnoveterinary medicine (EVMs) [21]. In Thailand, the bark is used in wound dressing, while the heartwood is utilized in remedies intended to soothe the throat and relieve thirst [23]. While the reviewed ethnomedicinal data is largely derived from secondary sources, it collectively points toward *L. coromandelica* as a multi‐purpose medicinal resource with promising pharmacological properties. The convergence of similar uses across diverse geographical areas strengthens the rationale for targeted phytochemical characterization and preclinical studies. Future research should prioritize standardizing extraction methods, bioactivity‐guided fractionation, and safety profiling to translate traditional knowledge into scientifically validated therapeutics.

## 5 Phytoconstituents

**Figure 3:**
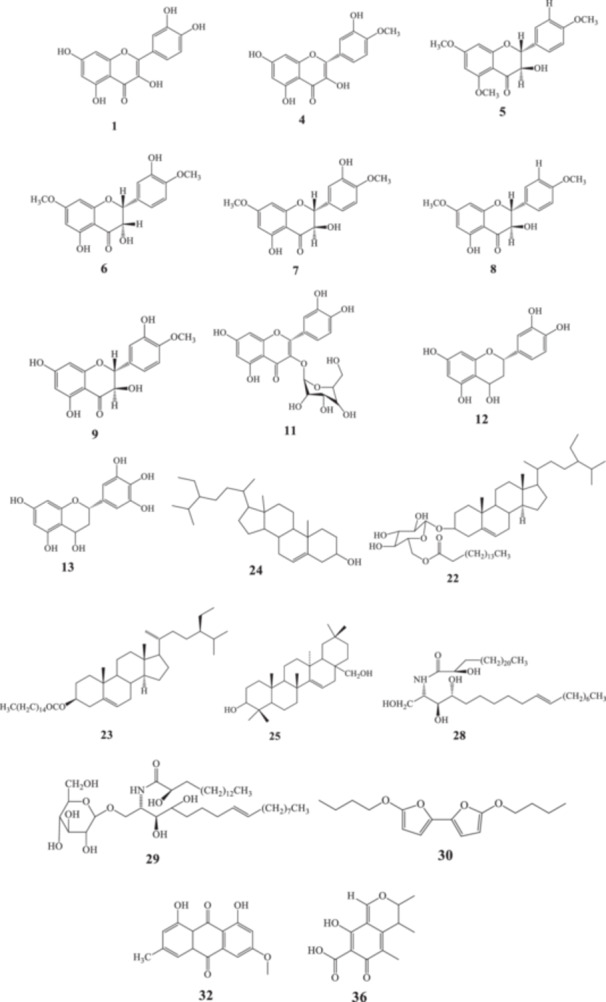
Chemical structures of major bioactive phytoconstituents identified in *L. coromandelica*.

Phytochemical studies of *L. coromandelica* have identified a diverse array of bioactive compounds across its leaves, bark, roots, stem, and fruits, predominantly including flavonoids, sterols, tannins, triterpenoids, and phenolic acids, which underpin its pharmacological activities. Approximately 36 distinct compounds have been reported and isolated from various plant parts [Table 3], including epicatechin, gallic acid, methyl gallate, β‐sitosterol, oleanolic acid, luteolin, and quercetin. These constituents exhibit therapeutic properties such as antioxidant, anti‐inflammatory, hepatoprotective, antidiabetic, and anticancer effects. The chemical structures of major compounds are illustrated in Figure 3, highlighting diverse functional groups and scaffolds. Gallic acid and methyl gallate, with multiple hydroxyl groups on aromatic rings, display potent free radical scavenging, while oleanolic acid, a pentacyclic triterpenoid with an oleanane skeleton, exhibits key anti‐inflammatory and hepatoprotective activities.

**Table 3:** A list of chemical compounds presents in *L. coromandelica*.

SI.	Class	Active compound	Parts	Extraction	Ref
1.	Flavonoids	Quercetin	Bark	Ethanol	[37]
			Leaves	Crude alcoholic	[13]
2.		Epigallocatechin‐3‐gallate (EGCG)	Bark	Methanol	[38]
3.		Catechin	Leaf		[5]
4.		Morin	Leaves, flower, stem bark	Crude alcoholic	[13]
5.		(2 R,3 R) ‐( + )‐4',5,7‐trimethoxydihydroflavonol	Stem bark	Crude alcoholic/acetone, n‐hexane, diethyl ether, ethyl acetate, methanol	[14]
6.		(2 R,3S)‐( + )‐3,5‐dihydroxy‐4,7‐dimethoxydihydroflavonol			
7.		(2 R,3 R)‐( + )‐4',7‐di‐O‐methyldihydroquercetin			
8.		(2 R,3 R)‐( + )‐4',7‐di‐O‐methyldihydrokaempferol			
9.		(2 R,3 R)‐( + )‐4'‐O‐methyldihydroquercetin			
10.	Flavanol glycoside	Quercetin‐3‐arabinoside	Flowers	Ether and ethyl acetate	[13]
			Leaves	Crude alcoholic	
11.		Isoquercetin	Flowers		
12.	Flavan‐3‐ol	Leucocyanidin	Leaves	Crude alcoholic	[13]
13.		Leucodelphinidin			
14.	Phenolic acid	Protocatechuic acid	Bark	Ethanol	[30]
15.		Gallic acid	Leaf	Methanol	[5]
16.	Phenolic aldehyde	Isovanillin	Bark	Ethanol	[37]
17.	Phenylpropanoid	Trans‐cinnamic acid	Bark	Ethanol	[37]
18.		Chlorogenic acid	Bark	Methanol	[38]
19.		Caffeic acid	Bark	Methanol	[38]
20.	Polyphenol	Ellagic acid	Leaf	Methanol	[5]
21.	Oestrogenic compound	*p*‐Hydroxybenzoic acid ethyl ester	Bark	Ethanol	[37]
22.	Sterol ester	β‐sitosteryl‐3β‐glucopyranoside‐6′‐O‐palmitate	Bark	Ethanol	[37]
23.	Phytosterol ester	Phytosterol‐β‐sitosterol palmitate	Bark	—	[14]
24.	Phytosterol	ß‐Sitosterol	Leaves	Crude alcoholic	[13]
			Flower, stem bark	—	[14]
25.	Triterpene	Myricadiol	Bark	Ethanol	[37]
26.	Saturated fatty acid	Palmitic acid			
27.		Stearic acid			
28.	Lipid derivative	(2S,3S,4 R,10E)‐2‐[(2 R)‐2‐hydroxy‐tetracosanoyl amino]‐10‐octadecene‐1,3,4‐triol			
29.	Glycosphingolipid	Aralia cerebroside			
30.	Organic compound	5,5‐Dibuthoxy‐2,2‐bifuran			
31.	Glycosides	Quercetin glycoside	Flowers	Ether and ethyl acetate	[13]
32.	Anthraquinone	Physcion	Leaves	Crude alcoholic	[39]
33.	Oligosaccharide	4‐O‐(α‐d‐galactopyranosyluronic acid)‐d‐galactose	Flower, stem bark	—	[14]
34.		6‐O‐(β‐d‐glucopyranosyluronic acid)‐d‐galactose		—	
35.		6‐O‐(4‐O‐methyl‐d‐glucopyranosyluronic acid)‐d‐galactose		—	
36.	Mycotoxin	Citrinin	Roots	Ethyl acetate	[33]

### 5.1 Flavonoids and Flavanol Glycosides

A wide spectrum of flavonoids **(1–9)** has been isolated from *L. coromandelica*, highlighting its rich phytochemical landscape. Quercetin (**1**) is found in both bark and leaves, extracted through ethanol and crude alcoholic solvents respectively [13, 37]. The bark is further enriched with Epigallocatechin‐3‐gallate (**2**), identified via methanolic extraction [38]. Leaf extracts reveal the presence of catechin (**3**), while multiple plant parts leaves, flowers, and stem bark yield morin (**4**) through crude alcoholic methods [13]. A distinctive group of methoxylated dihydroflavonols including compounds (**5–9**) has been predominantly isolated from the stem bark, emphasizing its unique role in housing complex flavonoid derivatives [14]. The presence of flavanol glycosides (**10–11**) further broadens the phytochemical profile. Quercetin‐3‐arabinoside (**10**) and its analogs are identified in flowers and leaves, using ether, ethyl acetate, and crude alcoholic extractions [13]. Isoquercetin (**11**) adds another dimension to the flower's flavonoid makeup, reinforcing the importance of floral parts as a glycoside source.

### 5.2 Flavan‐3‐ols, Phenolic Acids and Phenolic Aldehyde

Flavan‐3‐ols (**12–13**) contribute to the chemical diversity of the leaves. Leucocyanidin (**12**) and leucodelphinidin (**13**) were both isolated from crude alcoholic leaf extracts, showcasing the importance of this part in accumulating condensed tannin precursors [13]. Phenolic acids (**14–15**) appear across various parts. Protocatechuic acid (**14**) is derived from the bark using ethanol, while gallic acid (**15**) is found in the leaves via methanol extraction, adding to the plant's reservoir of hydroxybenzoic acids [5, 37]. Isovanillin (**16**) has been reported in bark extracts prepared with ethanol, representing the phenolic aldehyde class and highlighting another dimension of aromatic compound presence in the bark [30].

### 5.3 Phenylpropanoids, Polyphenols and Oestrogenic Compounds

Two key phenylpropanoids, trans‐cinnamic acid (**17**) and chlorogenic acid (**18**), have been isolated from bark using ethanol and methanol respectively [37, 38]. Caffeic acid (**19**), another member of this class, further reinforces the bark's role in concentrating aromatic secondary metabolites [38]. Ellagic acid (**20**), a representative polyphenol, was found in methanolic leaf extracts, contributing to the complex array of polyphenolic structures within the plant [5]. The oestrogenic compound p‐Hydroxybenzoic acid ethyl ester (**21**) has been identified from ethanol‐extracted bark samples, further diversifying the phenolic landscape [37].

### 5.4 Sterol, Phytosterol Esters, Phytosterols, and Triterpenes

Two sterol esters β‐sitosteryl‐3β‐glucopyranoside‐6'‐O‐palmitate (**22**) and phytosterol‐β‐sitosterol palmitate (**23**) were both isolated from bark extracts [14, 37]. These compounds underscore the bark's importance as a site of complex lipid‐derived phytoconstituents. β‐Sitosterol (**24**) has been identified in leaves, flowers, and stem bark, extracted via crude alcoholic methods and standard procedures [13, 14]. This widespread distribution signifies its abundant occurrence across aerial parts. Myricadiol (**25**), a triterpene, was isolated from the bark using ethanol, further contributing to the bark's phytochemical repertoire [37].

### 5.5 Saturated Fatty Acids, Lipid Derivatives, Glycosphingolipids and Organic Compounds

Palmitic acid (**26**) and stearic acid (**27**), both saturated fatty acids, were isolated from various plant parts, reflecting the plant's role in primary fatty acid production. A unique lipid derivative, (2S,3S,4 R,10E)‐2‐[(2 R)‐2‐hydroxy‐tetracosanoyl amino]‐10‐octadecene‐1,3,4‐triol (**28**), has been documented, showcasing the plant's potential for novel lipid structures. Aralia cerebroside (**29**), a glycosphingolipid, adds to the range of amphipathic molecules found in the plant, suggesting its diverse metabolic pathways. A notable organic compound, 5,5‐dibuthoxy‐2,2‐bifuran (**30**), was detected, hinting at unique heterocyclic structures within *L. coromandelica*.

### 5.6 Glycosides, Anthraquinones, Oligosaccharides and Mycotoxins

Quercetin glycoside (**31**) has been isolated from the flowers using ether and ethyl acetate extractions, showcasing the presence of flavonol‐based glycosides [13]. Physcion (**32**), an anthraquinone derivative, was found in the leaves from crude alcoholic extracts, highlighting the occurrence of aromatic ketone‐containing compounds [14, 39]. Oligosaccharides (**33–30**) were identified in flower and stem bark samples, indicating the presence of structurally diverse carbohydrate chains [14]. These include rare sugar acids like α‐d‐galactopyranosyluronic acid and 4‐O‐methyl‐d‐glucopyranosyluronic acid derivatives. Citrinin (36), a mycotoxin, has been reported in root extracts using ethyl acetate, revealing a rare case of fungal‐origin compounds associated with the plant's root microbiome or endophytic interactions [40].

## 6 Pharmacological Activity

**Figure 4:**
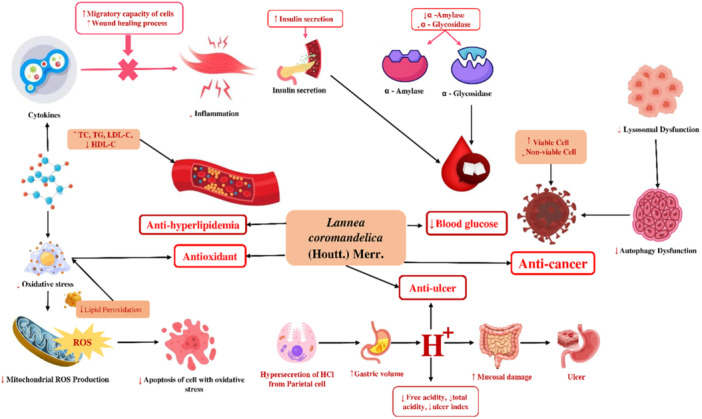
Overview of the diverse pharmacological effects of Lannea coromandelica and the proposed molecular and cellular mechanisms underlying its therapeutic activities.

*L. coromandelica* has been extensively investigated *in vitro*, and *in vivo* to validate its ethnomedicinal applications. Most studies have focused on its antidiabetic and cardioprotective activities, including molecular mechanisms of action of its extracts and phytoconstituents. Strong evidence also supports its anticancer, antibacterial, antimicrobial, antioxidant, and antitubercular potential. Preliminary studies indicate hypolipidemic, analgesic, anthelmintic, anti‐inflammatory, wound‐healing, antihyperuricemic, and antidiarrheal activities, though these remain underexplored and warrant further investigation. A concise summary of pharmacological activities is presented in Table 4, and mechanisms underlying various effects are illustrated in Figure 4.

**Table 4:** Pharmacological effects of *L. coromandelica* extracts observed in various experiments.

Activity	Parts	Preparation type/extract	Study type	Testing/study methods	Dose administration	Effects/result	Ref
Anti‐diabetic	Leaves	Methanol	*In Vivo*	Swiss albino mice, alloxan‐induced	200 and 400 mg/kg	Significant antidiabetic activties and ↓blood glucose levels	[41]
	Fruit	Methanol	*In vitro*	α‐glucosidase and α‐amylase	2, 4, 6, 8, and 10 mg mL − 1	↓ α‐glucosidase and α‐amylase activity	[32]
	Leaves	Ethanol	*In vivo*	Streptozocin‐induced diabetic rats	100 and 200 mg/kg	Antihyperglycemic, Antioxidant, and anti‐glycation properties that could ↓ the progression of diabetic nephropathy.	[42]
	Bark	Deionized water	*In Vitro*	HEK‐293 and HepG2	0.39, 0.78, 1.56, 3.13, 6.25, 12.5, 25 and 50 mg/m	Inhibit glucagon mediated cAMP formation and hyperglycemia in Type 2 diabetes.	[43]
	Bark	Methanol	*In Vivo*	Glucose‐loaded Swiss albino mice	100, 200, 400 mg/kg	↓ Serum glucose levels.	[44]
	Leaves	Water	*In Vivo*	Streptozocin‐induced diabetic wistar rats	50, 100, 150 mg.	No observed effect, likely due to low extract concentration.	[45]
Wound healing activity	Bark	Ethanol	*In Vivo*	Diabetic mice	100 mg	Healing diabetic wounds	[29]
Anticancer	Twigs	Ethanol	*In vitro*	HepG2 cells (human HCC cell line) and Vero cells	500 μg/mL	Moderate cytotoxicity	[5]
	Bark	Ethanol	*In Vitro*	B16F0 melanoma cell line	9.69 μg/mL	It exhibit potential anticancer effects	[46]
	Bark	Chloroform	*In vivo*	Male Swiss albino mice	200 mg/kg	Potential anticancer effects	[47]
	Stem Bark	Ethanol	*In vivo*	The white male rats of the Wistar strain	3150, and 3600 mg/kg.	↑ Mucosa and ↓ inflammatory cell infiltration	[36]
Antinociceptive	Leaves	Ethanol	*In vivo*	Chemical‐ and heat‐induced nociception in mice	50, 100, and 200 mg/kg	Antinociceptive effect via central and peripheral pathways, involving K⁺ channels and cGMP.	[48]
	Bark	Ethanol	*In vivo*	Swiss albino mice (male)	50, 100, and 200 mg/kg	Potential anti analgesic activity	[49]
	Bark	Methanol	*In vivo*	Albino mice, tail immersion method and acetic acid‐induced	200 and 400 mg kg^−1^	Potential antinociceptive agent	[18]
Antibacterial	Bark	Ethanol and distilled water	*In vitro*	*S. pyogens* (MTCC 1928), *S. aureus* (MTCC 3160), and fungi *C. albicans* (MTCC 183)	16, 12, and 8 mg	↑ antimicrobial, antifungal activity	[50]
	Fruit	Methanol	*In vitro*	*Proteus vulgaris, Klebsiella pneumoniae, Enterobacter aerogenes, Propionibacterium acnes, Staphylococcus aureus*, and *Streptococcus pyogene*s, agar (Mueller‐Hinton agar: MHA)	2.5, 5.0, 7.5, 10.0, 12.5, and 15.0 mg mL− 1	Potential antibacterial activity.	[51]
	Bark	Ethyl acetate and ethanol	*In vitro*	Diffusion method	350 g	All extracts showed antibacterial activity against *E. coli*, *S. aureus*, *Pseudomonas* sp., and *V. parahaemolyticus*	[52]
Antimicrobial	Leaf	Ethyl acetate	*In vitro*	Disc diffusion method	400 and 800 μg/disc	Moderate antimicrobial activity	[53]
	Bark					Weak antimicrobial activity	
	Bark	Chloroform	*In vitro*	Disc diffusion method	500 µg	Potential antimicrobial effects	[47]
	Bark	Methanol	*In vivo*	Rat	1000 μg mL^−1^	Potential antimicrobial effects	[54]
	Leaves and bark	Chloroform and methanol	*In vitro*	Disc diffusion method	250, 500 and 1000 μg	Methanol extracts exhibited stronger antimicrobial activity than chloroform extracts.	[55]
Antimalarial	Bark	Hexane and ethyl acetate	*In vitro*	*Plasmodium falciparum*	10, 1, 0.1, 0.01 and 0.001 µg/mL	Ethyl acetate isolate showed moderate, while hexane isolate showed low or negligible antiplasmodial activity	[56]
Antioxidant	Leaf and bark	Ethyl acetate	*In vitro*	DPPH Free radical scavenging	10 mg/m	Potential antioxidant activity and ↓ power	[53]
	Leaves	Methanol	*In vitro*	DPPH Radical scavenging	0–100 *μ*g/mL	It exhibits potential antioxidant activity	[5]
		Distilled water, ethyl acetate, ethanol 70%, and n‐hexane	*In‐vitro*	DPPH method by UV‐Vis spectrophotometry	40, 80, 120, 160, and 200 ppm	Strong antioxidant activity	[57]
		Ehyl acetate	*In vivo*	Wister rats, carbon tetrachloride (CCl_4_) induced oxidative toxicity	100, 200 and 400 mg/kg	It exhibits potential antioxidant activity and ↓the substances for the lipid peroxidation reaction product (MDA) in livers and kidney	[5]
	Bark	Chloroform	*In Vitro*	Phosphor molybdenum method	5 mg/mL	Potential antioxidant effects	[47]
		Methanol	*In vitro*	DPPH Radical scavenging	200 and 400 mg kg^−1^	Potential antioxidant effect	[38]
		Methanol	*In vitro*	RAW 264.7 cells and Skin fibroblast cells through DPPH radical‐scavenging assay	30 µg/mL	Exhibited strong antioxidant activity and ↓cellular ROS production.	
		Methanol and aqueous extract	*In vitro*	DPPH Radical scavenging	100–500 μL	Potential antioxidant effects	[58]
	Roots	Bleach solution, ethanol and distilled water	*In vitro*	DPPH Radical scavenging	200, 100, 50, 25, 12.5, 6.25 µg/mL	↓ Free radical′s activity	[40]
Antihistaminic	Leaves	Ethanol	*In vitro*	Isolated guinea pig Ileum	400 mg/kg	Inhibits histamine‐induced contraction and mast cell degranulation indicating its potential as a stabilizer	[59]
Antidiarrheal activity	Bark	Methanol	*In vivo*	Castor oil and magnesium sulphate induced diarrheal model, mice	100 and 200 mg/kg	Potential antibacterial effect	[60]
Anxiolytic	Leaves	Methanol	*In vivo*	Swiss albino mice	100, 150, and 200 mg/kg	↓Locomotor function, indicating CNS depressive properties	[41]
Anti‐ulcer	Leaves	70% Hydroethanolic mixture (water and ethanol)	*In vivo*	Albino wister rats in three models (stress induced, aspirin induced, and ulcer induced model)	250, 500 mg/kg	Significant levels of protection in ulcer	[61]
	Bark	Ethanol	*In vivo*	Aspirin – induced rats	400, 800, and 1200 mg/kg	↓ The ulcer index	[62]
Antithelmintic	Bark	Methanol and aquous	*In vitro*	Pheretima posthuma and Ascardia galli	10,25,50 mg/mL	Prominent anthelmintic properties.	[63]
Hypolipidemic activity	Leaves	Ethanol	*In vivo*	Triton X‐100 induced hyperlipidemic rats	100 and 200 mg/kg	Significant ↓in the total cholesterol levels	[64]

### 6.1 Antidiabetic Activity

Diabetes mellitus is a chronic metabolic disorder characterized by persistent hyperglycemia and impaired metabolism of carbohydrates, fats, and proteins. It arises from complete or partial insulin deficiency, reduced insulin sensitivity, or improper tissue response to insulin [65]. With the global rise in diabetes cases, numerous conventional antidiabetic drugs are available, particularly in industrialized nations; however, these treatments carry risks, including toxicity and renal complications [66]. Historical and traditional practices indicate that certain medicinal plants may serve as alternative or supportive therapies, even offering preventive benefits. Modern research is exploring bioactive plant compounds, such as antioxidants, for their therapeutic potential [67]. Various *in vitro* and *in vivo* studies have evaluated antidiabetic effects of leaves, bark, and fruits. Methanolic and ethanolic leaf extracts significantly reduced blood glucose in alloxan‐ and streptozotocin‐induced diabetic models, with methanolic extracts at 200–400 mg/kg lowering glucose in Swiss albino mice [41], while ethanolic extracts exhibited antioxidant, antihyperglycemic, and anti‐glycation effects at 100–200 mg/kg in diabetic rats [42]. Methanolic bark extracts demonstrated dose‐dependent reductions in serum glucose at 100–400 mg/kg [44], and aqueous bark extracts inhibited glucagon‐mediated cAMP in HEK‐293 and HepG2 cells [43]. Fruit extracts inhibited α‐glucosidase and α‐amylase at 2–10 mg/mL [51]. Some studies reported limited efficacy, such as aqueous leaf extract at 50–150 mg in diabetic Wistar rats [45], likely due to subtherapeutic dosing.

The detailed mechanisms by which *L. coromandelica* exerts its antidiabetic effects are illustrated in Figure 4.

### 6.2 Anticancer Activity

**Figure 5:**
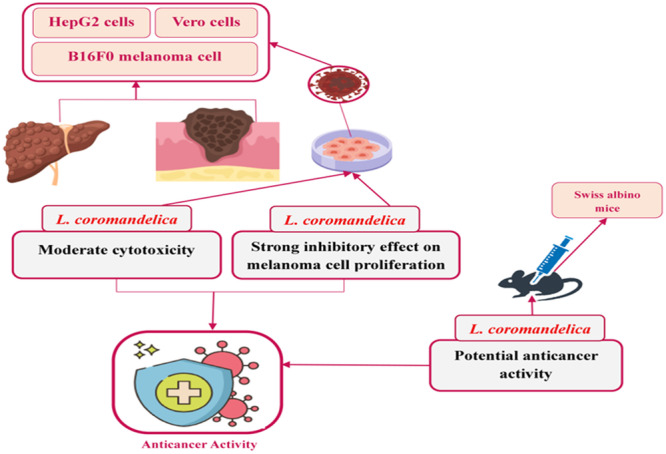
Proposed in vivo and in vitro effects of L. coromandelica in Anticancer activity.

Cancer arises when abnormal cells grow uncontrollably, with old cells surviving instead of undergoing programmed death, leading to the accumulation of abnormal cells [68]. It is the second leading cause of death worldwide, posing a major public health challenge. Standard treatments, including surgery, chemotherapy, radiation, and immunotherapy, often face limitations such as drug resistance and severe side effects, contributing to disease recurrence [69, 70]. Plant‐based drug development offers a promising alternative by leveraging the synergistic interactions of bioactive compounds in medicinal herbs with anticancer properties [71], Figure 5].

Preclinical studies have demonstrated the anticancer potential of various plant parts *in vitro* and in *vivo*. Ethanol twig extracts exhibited moderate cytotoxicity against HepG2 hepatocellular carcinoma cells at 500 μg/mL, with lower toxicity toward Vero cells, indicating selective activity [72]. Ethanol bark extracts showed potent effects against B16F0 melanoma cells at 9.69 μg/mL [46]. *In vivo*, chloroform bark extract administered at 200 mg/kg to male Swiss albino mice demonstrated systemic anticancer activity [47]. These findings highlight the cytotoxic and antitumor potential of bark and twigs, supporting their therapeutic promise as complementary or alternative cancer treatments, warranting further investigation into their mechanisms, active constituents, and clinical applications. The detailed mechanisms of *L. coromandelica′s* anticancer effects are illustrated in Figure 4.

### 6.3 Anti‐Inflammatory Activity

Inflammation is a critical defense mechanism that enables the body to respond to external threats, including injuries and pathogen‐induced infections, and plays an essential role in immune protection and tissue repair [73]. This complex biological process involves signaling molecules released by leukocytes, macrophages, and mast cells, along with complement activation, leading to fluid and protein leakage and accumulation of immune cells at the inflamed site [74]. While non‐steroidal anti‐inflammatory drugs (NSAIDs) effectively reduce pain and inflammation, they can cause adverse effects such as gastric ulcers and bleeding. Prolonged use of steroidal anti‐inflammatory drugs may affect the heart, hormonal balance, metabolism, bones, and eyes. In contrast, plant‐based therapies provide promising alternatives with fewer side effects [74, 75]. The bark of *L. coromandelica* exhibits significant anti‐inflammatory potential. Ethanol bark extracts, tested in carrageenan‐ and dextran‐induced edema in rats at 50–400 mg/kg, showed dose‐dependent inhibition of inflammation and leukocyte migration [61]. Additionally, stem bark ethanol extracts at 3150–3600 mg/kg in Wistar rats demonstrated gastroprotective effects, improving mucosal integrity and reducing inflammatory cell infiltration [36]. The detailed mechanisms are illustrated in Figure 4.

### 6.4 Antinociceptive Activity

Several *in vivo* studies have highlighted the promising antinociceptive effects of different plant parts using various extraction methods. Ethanolic leaf extracts, tested on mice under chemical‐ and heat‐induced nociception at 50, 100, and 200 mg/kg, demonstrated significant peripheral and central analgesic mechanisms, involving ATP‐sensitive potassium channels and the cGMP pathway [48]. Similarly, ethanolic bark extract, evaluated in Swiss albino male mice at the same doses, exhibited notable central analgesic activity [49]. Methanolic bark extract, tested at 200 and 400 mg/kg via tail immersion and acetic acid‐induced writhing tests, showed potent antinociceptive effects, supporting its role as an effective pain‐relieving agent [76].

### 6.5 Antibacterial Activity

The plant exhibits notable antibacterial and antifungal potential across various *in vitro* studies using multiple parts and solvent extracts. Ethanolic and aqueous bark extracts, tested against *Streptococcus pyogenes* (MTCC 1928), *Staphylococcus aureus* (MTCC 3160), and *Candida albicans* (MTCC 183) at 100% (16 mg), 75% (12 mg), and 50% (8 mg), showed strong antimicrobial activity, supporting traditional use in treating female reproductive tract infections [50]. Methanolic fruit extract demonstrated broad‐spectrum antibacterial effects against *Proteus vulgaris*, *Klebsiella pneumoniae*, *Enterobacter aerogenes*, *Propionibacterium acnes*, *Staphylococcus aureus*, and *Streptococcus pyogenes* at 2.5–15 mg/mL [51]. Further bark studies using ethanolic and ethyl acetate extracts confirmed activity against *Escherichia coli, Staphylococcus aureus, Pseudomonas spp*., and *Vibrio parahaemolyticus* [52].

### 6.6 Antimicrobial Activity

The antimicrobial potential of various plant parts has been extensively assessed using diverse extraction methods and experimental models. Ethyl acetate leaf extracts, evaluated via the disc diffusion method at 400 and 800 µg/disc, displayed moderate activity, whereas bark extracts were comparatively weaker [53]. Chloroform bark extracts, tested at 500 µg/disc, showed promising antimicrobial activity [47]. *In vivo* studies confirmed these results, with methanolic bark extract demonstrating significant antimicrobial effects in rats at 1000 µg/mL [54]. Combined methanolic extracts of leaves and bark, applied at 250, 500, and 1000 µg, exhibited stronger antimicrobial activity than chloroform extracts [55].

### 6.7 Antioxidant Activity

During ATP production, oxygen reduction generates reactive oxygen species (ROS), including hydroxyl and superoxide radicals. Although physiologically important, excessive ROS accumulation disrupts redox balance, damages biomolecules, and compromises cellular stability [77]. Free radicals are implicated in the pathogenesis of cancer, Alzheimer's disease, Parkinson's disease, cardiovascular disorders, diabetes, inflammatory conditions, stroke, and lipid peroxidation, highlighting their role in oxidative stress–related diseases [78]. Antioxidants counteract this imbalance by neutralizing ROS and protecting cells from oxidative injury [79]. Among them, plant‐derived antioxidants are particularly valued for their free radical–scavenging capacity and reducing potential [80].

*L. coromandelica* exhibits notable antioxidant activity across multiple experimental models. Ethyl acetate extracts of leaves and bark showed the highest scavenging power at 10 mg/mL in DPPH assays [53], while methanol leaf extracts displayed strong activity over 0–100 μg/mL [5]. Additional assays using distilled water, ethyl acetate, ethanol (70%), and n‐hexane extracts confirmed potent free radical scavenging at 40–200 ppm [57]. *In vivo*, ethyl acetate extracts (100–400 mg/kg) significantly mitigated CCl₄‐induced oxidative toxicity in Wistar rats, enhancing superoxide dismutase (SOD), catalase (CAT), and glutathione peroxidase (GSH‐Px) activity, while lowering malondialdehyde (MDA) levels in hepatic and renal tissues [5]. Bark extracts also demonstrated strong antioxidant potential, with chloroform fractions showing reducing capacity in the phosphomolybdenum assay at 5 mg/mL [47], and methanol fractions exhibiting DPPH scavenging at 200–400 mg/kg [76]. Methanolic extracts further reduced ROS generation in RAW 264.7 macrophages and fibroblasts at 30 µg/mL [38], while aqueous and methanol extracts showed dose‐dependent DPPH activity at 100–500 µL [58]. Root extracts prepared with bleach, ethanol, and water displayed robust antioxidant effects across 6.25–200 µg/mL [40]. The mechanisms underlying these effects are illustrated in Figure 4.

### 6.8 Anti‐Ulcer Activity

Peptic ulcer disease refers to mucosal lesions occurring in the stomach or proximal small intestine, commonly associated with excessive gastric acid secretion, weakened mucosal defense, or microbial infections [81, 82]. Standard therapies include non‐steroidal anti‐inflammatory drugs (NSAIDs), proton pump inhibitors (PPIs), histamine H2 receptor antagonists (H2RAs), and cytoprotective agents, but these often carry drawbacks such as mucosal injury, incomplete acid suppression, and adverse drug interactions [83]. Consequently, attention has shifted toward medicinal plants, which are historically valued for their complex bioactive compounds. While ethnobotanical use provides a compelling rationale, rigorous regulatory oversight and well‐designed clinical trials remain necessary to ensure safety and efficacy in ulcer management [84].

*L. coromandelica* has shown notable gastroprotective effects, particularly in experimental models employing leaf and bark extracts. A 70% hydroethanolic leaf extract demonstrated significant protection in stress, aspirin, and other ulcer‐inducing in Albino Wistar rats. Both low and high doses offered benefit, with the higher dose showing superior efficacy. In aspirin‐induced models, the extract achieved ulcer inhibition comparable to ranitidine, highlighting its therapeutic relevance [61]. Likewise, ethanol bark extracts markedly reduced ulcer indices in aspirin‐treated rats, with protective effects observed at 400, 800, and 1200 mg/kg [62]. These findings collectively indicate that *L. coromandelica* holds promise as a natural anti‐ulcer agent. The mechanistic basis for these protective actions is further detailed in Figure 4.

### 6.9 Other Pharmaceutical Activities

Several studies have revealed the diverse pharmacological activities of various parts of the *L. coromandelica*, highlighting its potential in modern therapeutics. The ethanol extract of the bark demonstrated significant wound healing effects in diabetic mice at a dose of 100 mg, suggesting its usefulness in treating chronic diabetic wounds [34]. In terms of anti‐malarial activity, ethyl acetate and hexane extracts of the bark were tested against *Plasmodium falciparum*, where the ethyl acetate isolate showed moderate efficacy, while the hexane fraction exhibited minimal or no activity [56]. Ethanol extracts of the leaves showed notable antihistaminic properties by inhibiting histamine‐induced contraction and mast cell degranulation in isolated guinea pig ileum, indicating its potential as a mast cell stabilizer [59]. In antidiarrheal tests, methanol extracts of the bark significantly inhibited castor oil and magnesium sulphate‐induced diarrhea in mice, showing the highest effectiveness against *Escherichia coli* [60]. Methanol extracts of the leaves exhibited anxiolytic activity in Swiss albino mice, marked by decreased locomotor function, indicating central nervous system depressant effects [41]. Additionally, the methanol extract of the bark showed potent anthelmintic effects against *Pheretima posthuma* and *Ascardia galli*, outperforming the aqueous extract [63]. Finally, ethanol extracts of the leaves displayed hypolipidemic activity in Triton X‐100‐induced hyperlipidemic rats, significantly reducing total cholesterol levels at doses of 100 and 200 mg/kg [64]. The detailed mechanisms by which *L. coromandelica* exerts its antihyperlipidemic effects are illustrated in Figure 4.

## 7 Safety Profile

*L. coromandelica* has consistently demonstrated a favorable safety profile across *vivo* models. Oral administration of its ethanolic extract at doses between 500 and 2500 mg/kg caused no observable behavioral changes, allergic reactions, or mortality in animal studies [49]. Similarly, the methanolic extract, selected for its antibacterial efficacy, showed no significant toxic effects in acute toxicity evaluations, with vital organ functions remaining unaffected [51]. Biochemical parameters including serum albumin (5.1 g/dL), bilirubin (0.62 g/dL), and total protein (7.5 g/dL) were all within physiological limits, confirming preserved hepatic and renal integrity. Animals displayed normal activity, grooming, gait, and sensory responses, with no signs of depression, lacrimation, stimulation, or salivation [42]. Furthermore, oral administration of ethanolic extract (EELC) at 1000–3000 mg/kg produced no adverse outcomes, reinforcing its low toxicity and confirming an LD₅₀ above 3000 mg/kg [48]. Likewise, oral delivery of *L. coromandelica* gum at 2000 mg/kg in mice resulted in no visible toxic effects or mortality [85]. Collectively, these findings highlight a high safety margin; however, comprehensive chronic toxicity studies, drug interaction assessments, and regulatory clarifications remain essential for clinical translation [86].

## 8 Future Perspective and Conclusion

Our synthesis of the current literature reveals both the remarkable promise and the striking gaps in *L. coromandelica* research. While a wide spectrum of pharmacological activities has been reported, the evidence base remains heavily weighted toward preliminary *in vitro* and small‐scale *in vivo* models. Notably, several studies report dose‐dependent bioactivity, yet lack reproducibility across laboratories, suggesting methodological inconsistency in extraction protocols and bioassays. Furthermore, most pharmacological evaluations fail to correlate observed effects with specific phytoconstituents, leaving the mechanistic pathways largely speculative. A critical observation is the uneven exploration of plant parts: bark and leaves dominate existing studies, whereas gum, roots, and fruits traditionally valued in ethnomedicine remain neglected. This imbalance limits a complete understanding of the plant's therapeutic spectrum. Equally concerning is the absence of chronic toxicity and pharmacokinetic studies, claims of safety based solely on acute models are insufficient for translational relevance. The isolation of individual compounds, such as quercetin and oleanolic acid, has demonstrated potential, but the synergistic or antagonistic interactions within the phytocomplex remain underexplored, risking oversimplification of its pharmacology. Another striking gap is the disconnection between ethnomedicinal claims and modern validation. For instance, despite extensive traditional use in skin and reproductive disorders, rigorous pharmacological studies in these domains are scarce. Bridging such gaps will not only substantiate traditional knowledge but also guide rational drug development. In conclusion, *L. coromandelica* offers genuine therapeutic promise, but without mechanistic clarity, rigorous standardization, and safety validation, its progression from folklore to evidence‐based medicine will remain incomplete.

## Author Contributions

**Nawfal Hasan Siam:** conceptualization, supervision, formal analysis, project administration, writing – review and editing, writing – original draft, methodology, investigation, funding acquisition, resources. **Sumaya Akter:** writing – original draft, data curation, resources. **Anika Rahman:** writing – original draft, resources, data curation. **Taniza Tasnim:** writing – original draft, resources, data curation. **Md. Mhamudul Huda Ruhed:** writing – original draft, resources, data curation. **Anika Ahmed:** writing – original draft, data curation, resources. **Naznin Rahman:** writing – original draft, data curation, resources. **Afsana Akter Mim:** writing – original draft, data curation, Resources. **Tasnim Tabassum:** writing – original draft, data curation, resources. **Md. Mazharul Islam Chowdhury:** writing – review and editing, supervision, formal analysis. **Jakir Ahmed Chowdhury:** writing – review and editing, supervision, formal analysis.

## Funding

The authors received no specific funding for this work.

## Ethics Statement

All authors have read and approved the final version of the manuscript [Corresponding author or manuscript guarantor] had full access to all of the data in this study and takes complete responsibility for the integrity of the data and the accuracy of the data analysis.

## Conflicts of Interest

The authors declare no conflicts of interest.

## 1 Transparency Statement

The lead author Nawfal Hasan Siam affirms that this manuscript is an honest, accurate, and transparent account of the study being reported; that no important aspects of the study have been omitted; and that any discrepancies from the study as planned (and, if relevant, registered) have been explained.

## Data Availability

The authors have nothing to report.
